# Direct and Indirect Effects of Climate Change on a Prairie Plant Community

**DOI:** 10.1371/journal.pone.0006887

**Published:** 2009-09-03

**Authors:** Peter B. Adler, James Leiker, Jonathan M. Levine

**Affiliations:** 1 Department of Wildland Resources and the Ecology Center, Utah State University, Logan, Utah, United States of America; 2 Research Technician, Kansas State University, Research and Extension, Hays, Kansas, United States of America; 3 Department of Ecology, Evolution and Marine Biology, University of California, Santa Barbara, California, United States of America; Umea University, Sweden

## Abstract

**Background:**

Climate change directly affects species by altering their physical environment and indirectly affects species by altering interspecific interactions such as predation and competition. Recent studies have shown that the indirect effects of climate change may amplify or counteract the direct effects. However, little is known about the the relative strength of direct and indirect effects or their potential to impact population persistence.

**Methodology/Principal Findings:**

We studied the effects of altered precipitation and interspecific interactions on the low-density tiller growth rates and biomass production of three perennial grass species in a Kansas, USA mixed prairie. We transplanted plugs of each species into local neighborhoods of heterospecific competitors and then exposed the plugs to a factorial manipulation of growing season precipitation and neighbor removal. Precipitation treatments had significant direct effects on two of the three species. Interspecific competition also had strong effects, reducing low-density tiller growth rates and aboveground biomass production for all three species. In fact, in the presence of competitors, (log) tiller growth rates were close to or below zero for all three species. However, we found no convincing evidence that per capita competitive effects changed with precipitation, as shown by a lack of significant precipitation × competition interactions.

**Conclusions/Significance:**

We found little evidence that altered precipitation will influence per capita competitive effects. However, based on species' very low growth rates in the presence of competitors in some precipitation treatments, interspecific interactions appear strong enough to affect the balance between population persistence and local extinction. Therefore, ecological forecasting models should include the effect of interspecific interactions on population growth, even if such interaction coefficients are treated as constants.

## Introduction

Climate change directly affects species by altering their physical environment and indirectly affects species by altering interspecific interactions such as predation and competition [1]–[2]. These indirect effects may amplify or counteract the direct effects of climate change. For example, negative direct effects of warming on a plant species may be offset if warming sufficiently reduces the abundance of that plant's enemies. Recent empirical work has shown that indirect effects mediated by interspecific interactions can be as or more important than the direct effects of climate change [2]–[6], [but see 7].

Climate change could alter interspecific interactions in two distinct ways. First, climate change may influence the absolute or relative abundance of a species' competitors, predators, and pathogens. Second, climate change could alter the per capita effects of these heterospecifics on the focal species. A simple population growth rate equation illustrates these two possibilities for competitive systems:
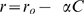
(1)
*r* is the realized population growth rate of a focal species, *ro* is the species' intrinsic rate of increase under a particular climate, α is a competition coefficient describing the per capita effect of competitors on the focal species, and *C* represents the abundances of heterospecific competitors. The direct effects of climate change alter organism performance via *ro*, while the indirect effects mediated by competitors emerge from changes in the second term. Changes in *C* arise from short term changes in the abundances of species already present in the community as well as longer term changes caused by local extinctions and immigration of new species [8]. Changes in α are most likely when climate change alters the resources for which species compete. Following Wootton [9], indirect effects caused by changes in *C* could be regarded as “chains of direct interactions” while indirect effects caused by changes in α would be “interaction modifications.”

Climate change could exert indirect effects through both of these pathways. Suttle et al. [5] found that California grassland forbs declined with increased precipitation due to the favorable response of their annual grass competitors. In this case, indirect effects were driven primarily by changes in *C*, competitor abundance. In contrast, plant ecologists have demonstrated that α, the per capita effect of neighbors, may respond to the abiotic environment. For example, in high resource areas, interspecific interactions are primarily competitive, but competition may give way to facilitation under the most stressful conditions [10]–[11]. While climate change could alter both α and *C*, we might expect changes in C to occur over longer time scales. Determining how these mechanisms will impact population growth relative to the direct effects of climate change will be essential for predicting where and when indirect effects of climate change will be most important.

The potential for climate change to drive species to local extinction is of particular conservation concern. Evaluating the impact of either direct or indirect effects on population persistence requires experiments that quantify a focal species' population growth when it is rare and its competitors are common. This is why equation (1) ignores conspecific density entirely, which is appropriate when the focal species is rare. If this growth rate is positive (on average), the species will tend to persist [12], whereas a negative growth rate when rare predicts local extinction. Unfortunately, few competition experiments are designed to measure growth rates when rare. One problem is that many fitness measures, such as size, may be poor proxies for the population growth rate. A second problem is that measuring the growth rate when rare requires a community in which the focal species is at low abundance while its competitors' abundances are close to their stochastic equilibrium. If the focal species is currently at high abundance, determining the appropriate abundances of the resident species may be difficult (but see [13]–[14]).

We used an experimental approach to study the effects of precipitation and interspecific interactions on three perennial grass species in a Kansas, USA mixed prairie. Future changes in precipitation regimes, which remain uncertain for this region [15]–[16] could exacerbate or ameliorate water limitation [17]. We transplanted plugs of the three study species into local neighborhoods of heterospecific competitors and then exposed the plugs to a factorial manipulation of growing season precipitation and neighbor removal. Our experiment is novel because it evaluates the tiller and biomass growth of the focal grasses at the low densities which determine persistence.

In our one-year study, the precipitation manipulations had little time to alter the abundances or identities of the resident competitors, making indirect effects mediated via changes in *C* unlikely. Instead, our experiment focuses on whether changes in precipitation alter the per capita effects of competition, α. We addressed two research questions about indirect effects of climate change. First, do the effects of competition change across the experimental precipitation treatments, as shown by the precipitation × competition interaction in our statistical models? Second, how strong are indirect effects relative to the direct effects of climate change? Our third research question concerns the combined effects of precipitation change, direct and indirect, on population persistence: What is the potential for a change in precipitation to cause negative low density growth rates?

## Methods

### Site description

The study site is located 3.5 km west of Hays, KS (38.8°N, 99.3°W). Mean annual temperature is 12°C and mean annual precipitation is 580 mm, 75% of which falls in spring and summer. We conducted the experiment on shallow limestone soils dominated by three perennial warm season grasses, *Bouteloua curtipendula, Bouteloua hirsuta*, and *Schizachyrium scoparium* (the plant community is described by [18] and [19]). The pasture, which has never been cultivated, is grazed at light to moderate intensity by livestock during spring and summer. We used electrical fence to exclude livestock from the study plots throughout the experiment.

### Experimental design

In April, 2007 we located two blocks of 9 plots separated by 0.5 km. Each plot is 8 m long, oriented with the slope, and 2 m wide. In May and June of 2007 we transplanted 6 plugs of each of the 3 study species into each plot (yielding 18 total plugs per plot). A suitable transplant location was defined as a circular neighborhood of radius 20 cm in which the target species did not occur, allowing us to assess its performance when rare. In other words, each transplant will experience heterospecific but not conspecific competition, though the abundances of the heterospecific neighbors are not controlled. For each transplant, we first removed a soil core, 5 cm wide and 10 cm deep, at its target location. Second, we extracted a similarly sized core containing a plug of the target species and inserted it into the target hole. We transplanted 324 plugs in total (6 plugs of each of the three species in each of the 18 replicate plots). We spot-watered the plugs as necessary during the 2007 growing season and replaced dying plugs before mid-July. Precipitation was above-normal in the 2007 water year (817 mm), contributing to a high success rate for the transplants. In September 2007, we counted the number of live tillers of each plug.

Before the 2008 growing season, we randomly assigned the plots to one of three precipitation treatments: drought, ambient, and irrigated (6 replicates of each treatment). The purpose of the treatments was to create large differences in growing season precipitation, rather than to simulate a particular future precipitation forecast. We imposed drought using passive 10 m long ×4 m wide rainfall shelters that intercept approximately 50% of incoming rainfall [20] beginning in late March 2008. The pitched roofs of the shelters were made of 15 cm wide strips of corrugated polycarbonate with >90% PAR transmittance (Dynaglass brand) which channeled rainfall into gutters that lead away from the plots. Rain falling between the roofing strips reaches the plot. No transplant plugs were placed within a 1 m buffer inside the edge of the shelter.

We applied water to the irrigation treatment by pumping water from a 1500 gallon holding tank and into a network of soaker hoses [21]. We used municipal water low in nitrates. Each week from May through September we applied the long-term average weekly precipitation. This “ambient + normal” approach ensured a wetter than normal treatment, even if ambient precipitation was well below normal. To compensate for a 3 week interruption to our normal irrigation schedule in June, we increased the July watering totals. In two plots of each treatment we monitored soil moisture and air temperature, logging observations every 15 minutes. Volumetric soil moisture was measured with Decagon Devices EC-5 probes.

Based on a May 2008 tiller census, we discarded 31 transplants due to small size (6 *B. curtipendula*, 8 *B. hirsuta*, 17 *S. scoparium*). For the remaining plugs, we randomly assigned a neighbor removal treatment to 2–3 plugs (depending on the surviving sample size) of each species in each replicate plot. The neighbor removal treatment consisted of clipping all competing vegetation within a 20 cm radius of the target plug and then treating with herbicide.

We conducted a final tiller count in September, 2008, and counted vegetative and reproductive tillers separately. Finally, we harvested all aboveground biomass of the transplants. An additional 17 transplants, fairly evenly distributed among all three species, were removed from the analysis due to disturbance from digging animals (12 transplants) or mortality (5 transplants). For each surviving transplant, we calculated a population growth rate as the log of the proportional change in tiller number from September 2007 to September 2008. Although this population growth rate (or tiller growth rate) ignores seedling recruitment, the growth of long-lived perennial populations is typically driven by survival and growth rather than reproduction [22].

### Statistical analysis

For each species, we analyzed tiller growth rates as a function of precipitation treatment, neighbor removal (nested within a random effect for plot), and their two-way interaction, using a linear mixed-effect model (the “lme” function of package nlme in R 2.8 [23]). We calculated each transplant's tiller growth rate as log(September 2008 tillers)-log(September 2007 tiller), including both vegetative and reproductive tillers. The log transformation ensures that decreases and increases in tiller numbers are weighted equally. We did not include a random block effect after determining that block did not explain significant variation among treatments. We used a similar model to analyze log-transformed aboveground biomass, but incorporated the September 2007 tiller count as a covariate.

## Results

### Treatment environmental effects

**Figure 1:**
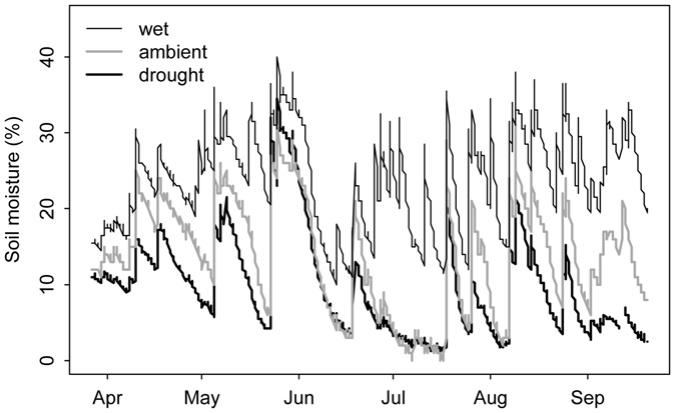
Effects of precipitation treatments on volumetric soil moisture during the 2008 growing season for each of the three precipitation treatments. Data are from representative plots in the north block.

The precipitation treatments succeeded in creating large differences in soil moisture among the plots (Fig. 1). For the 2008 water year, the drought plots received 498 mm, the ambient plots received 816 mm, and the irrigated treatments received 1139 mm. The rainfall shelters had little effect on average daily temperatures, but did reduce thermal amplitude: Maximum temperatures were about 2°C lower than in ambient plots, and minimum temperatures were about 2°C higher.

### Plant responses

**Figure 2:**
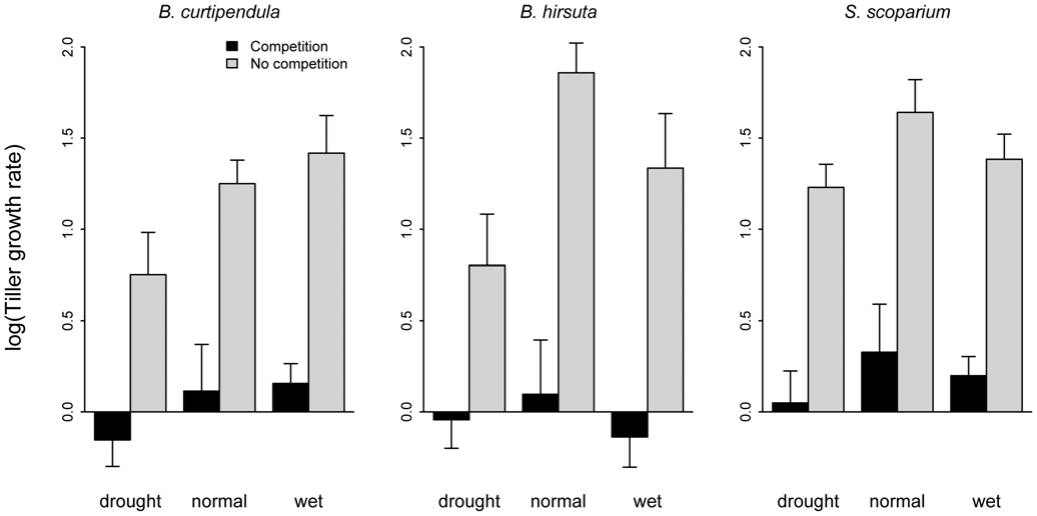
Mean log tiller growth rates of the transplants by species and precipitation treatment. Bars show empirical standard errors. Table 1 contains full statistical results.

For all three species, the presence of competitors had a significant negative effect on tiller growth rates when rare, generally reducing log growth rates from 1.5–2 to near zero (Fig. 2, Table 1). Both *Bouteloua* species had at least one precipitation treatment mean that fell below zero, and *S. scoparium*'s mean in the drought treatment fell within one standard error of zero (Fig. 2). The main effect of precipitation was significant for one of three focal species, *B. curtipendula*, roughly doubling growth in the irrigated treatment relative to the drought treatment. Precipitation did not alter the effect of competition on any focal species, as shown by non-significant precipitation × competition interactions (Table 1).

**Table 1:** Analysis of variance results for tiller growth rates.

	DF	F-value	p-value
*B. curtipendula*			
Precipitation	2,15	5.423	0.0169
Competition	1,15	62.193	<0.001
Precipitation×Competition	2,15	0.526	0.6014
*B. hirsuta*			
Precipitation	2,15	2.118	0.1548
Competition	1,13	66.192	<0.001
Precipitation×Competition	2,13	2.338	0.1357
*S. scoparium*			
Precipitation	2,15	2.097	0.1573
Competition	1,14	47.688	<0.001
Precipitation×Competition	2,14	0.099	0.9060

**Figure 3:**
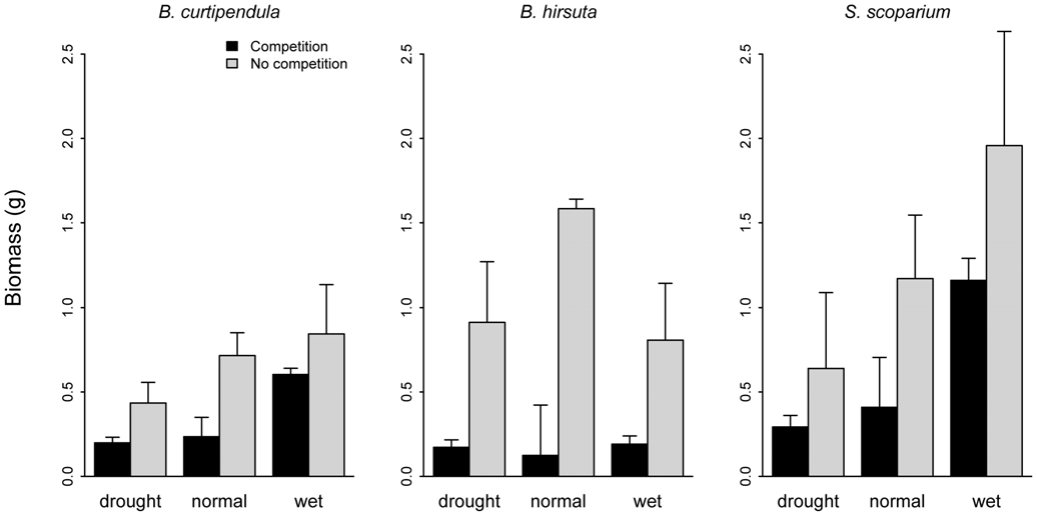
Mean aboveground biomass of the transplants by species and treatment. Bars show empirical standard errors. Table 2 contains full statistical results.

Shoot biomass increased significantly with competitor removal for two of three focal species, and approached significance for the third (*S. scoparium*) (Fig. 3, Table 2). In each case, removal of competitors more than doubled biomass (Fig. 3, Table 2). Two of three species (*B. curtipendula S. scoparium*) also showed a significant response to precipitation, producing about two-fold more biomass in the irrigated treatment than in the drought treatment (Fig. 3, Table 2). For *B. hirsuta*, precipitation did not have a significant effect on biomass, but the precipitation × competition interaction was significant (Fig. 3, Table 2). However, for this species, the largest effect of competition occurred in the ambient rather than the irrigated treatment.

**Table 2:** Analysis of covariance results for biomass, with tiller numbers as the covariate.

	DF	F-value	p-value
*B. curtipendula*			
Precipitation	2,15	5.824	0.0134
Competition	1,14	15.517	0.0015
Tiller number	1,14	4.595	0.0501
Precipitation×Competition	2,14	1.676	0.2226
*B. hirsuta*			
Precipitation	2,15	0.310	0.7383
Competition	1,11	47.949	<0.001
Tiller number	1,11	12.045	0.0052
Precipitation×Competition	2,11	4.018	0.0490
*S. scoparium*			
Precipitation	2,15	4.140	0.0370
Competition	1,13	3.550	0.0821
Tiller number	1,13	14.541	0.0022
Precipitation×Competition	2,13	1.861	0.1947

## Discussion

### Direct and indirect effects of precipitation change

For two of the three focal species, we found significant main effects of precipitation on tiller growth rate or biomass. In each of these cases, additional rainfall increased tiller or biomass growth while decreased rainfall reduced performance. These results indicate that future changes in precipitation, whether increases or decreases, will directly affect the performance of the grasses we studied.

In contrast, we found little evidence that precipitation change will modify interspecific interactions. For all three species, the presence of competitors had a strong negative affect on tiller growth rates, but the strength of the effect did not change with the precipitation treatment. Results for biomass growth were similar, although for one species, *B. hirsuta*, a significant precipitation×competition emerged. In this case, however, competition was actually stronger in the ambient than drought or irrigation treatments. This result is not easily reconciled with current thinking about competition along stress gradients [11]. The lack of convincing precipitation×competition interactions in our experiment, coupled with significant direct effects, provides clear answers to our first two research questions: changes in precipitation are unlikely to alter per capita competitive effects (α in equation 1) in this grassland community, and direct effects of climate change will have relatively stronger impacts on species' performance.

Our short-term study, which examined the effect of the current resident community on the focal species, did not address indirect effects caused by changes in competitor abundance or identity (*C* in equation 1). Over longer time scales, changes in precipitation regimes will alter relative abundances, allow new species to colonize, and cause the local extirpation of current members of the community [8]. In fact, the trends in our data suggest that the three study species would respond in different ways to an increase in rainfall (Fig. 2, 3). Changes in community composition could alter the net effect of competition on focal species, even if the α's remain constant. We could test such effects by maintaining the precipitation treatments for a number of years, allowing changes in community composition to occur, and then repeating the transplant experiment.

A caveat to our conclusion that precipitation will not modify per capita competitive effects is that we conducted our experiment during two very wet years. Ambient precipitation was 815–820 mm in the year of our precipitation manipulations and in the preceding year. As a result, our “drought” treatment of 498 mm was only modestly below the average annual precipitation of 580 mm. Meanwhile, our ambient (816) and irrigation treatments (1139) were well above average. It is possible that under true drought conditions we would have found a weakening of aboveground competition or some facilitation. However, such a finding would require strong non-linearity in how these species respond to precipitation.

### Precipitation change and population persistence

Although we found little evidence that precipitation can modify competitive interactions, the combined effects of precipitation and competition were sufficient to cause some species to experience negative low density growth rates, implying a trajectory to local extirpation. Given that competition in this system is strong enough to reduce these growth rates near zero, even small changes in plant performance due to the direct effects of altered precipitation change could threaten long-term persistence.

Our analysis of low density growth rates are based on transplanted plugs, which may not be representative of natural plants. For example, if our transplants are less robust than established mature plants, our estimates of tiller growth rates might be artificially low. However, our transplanted plugs showed high growth rates in the absence of competition and their mortality rates were low, indicating high vigor. In addition, the use of soil cores for transplanting may have given them some relief from competition by severing neighbors' roots. Therefore, whether our plugs perform better or worse than their natural equivalents is difficult to predict. Our analysis also assumes that all population growth is asexual tiller growth. Although sexual reproduction undoubtedly occurs, the population growth of long-lived perennial plants is typically driven by survival and growth, not reproduction [22]. It is hard to imagine that population growth as a whole could be strongly positive under conditions that caused small but established individuals to experience negative tiller growth rates.

### Conclusions

Our results have two implications for attempts to forecast the effect of climate change on biodiversity. First, because we found little evidence that altered precipitation will modify per capita competitive effects, indirect effects of climate change are more likely to be caused by changes in the composition of the resident community. Second, based on species' low growth rates when rare in the presence of competitors, interspecific interactions appear strong enough to affect the balance between population persistence and local extinction under some climate change scenarios. Therefore, ecological forecasting models should include the effect of interspecific interactions on population growth, even if such interaction coefficients are treated as constants. Finally, our experiment demonstrates how quantifying low density population growth rates addresses the population dynamic consequences of altered interspecific interactions under climate change.
